# 20-HETE promotes glucose-stimulated insulin secretion in an autocrine manner through FFAR1

**DOI:** 10.1038/s41467-017-02539-4

**Published:** 2018-01-12

**Authors:** Sorin Tunaru, Remy Bonnavion, Isabell Brandenburger, Jens Preussner, Dominique Thomas, Klaus Scholich, Stefan Offermanns

**Affiliations:** 1 Max Planck Institute for Heart and Lung Research, Department of Pharmacology, Ludwigstr. 43, 61231 Bad Nauheim, Germany; 2 ECCPS Bioinformatics Facility, Max Planck Institute for Heart and Lung Research, Ludwigstr 43, 61231 Bad Nauheim, Germany; 3 Institut für Klinische Pharmakologie, Pharmazentrum Frankfurt, ZAFES, Klinikum der Goethe-Universität Frankfurt, 60590 Frankfurt, Germany; 4 Centre for Molecular Medicine, Medical Faculty, J.W. Goethe University Frankfurt, Theodor-Stern-Kai 7, 60590 Frankfurt, Germany

## Abstract

The long-chain fatty acid receptor FFAR1 is highly expressed in pancreatic β-cells. Synthetic FFAR1 agonists can be used as antidiabetic drugs to promote glucose-stimulated insulin secretion (GSIS). However, the physiological role of FFAR1 in β-cells remains poorly understood. Here we show that 20-HETE activates FFAR1 and promotes GSIS via FFAR1 with higher potency and efficacy than dietary fatty acids such as palmitic, linoleic, and α-linolenic acid. Murine and human β-cells produce 20-HETE, and the ω-hydroxylase-mediated formation and release of 20-HETE is strongly stimulated by glucose. Pharmacological inhibition of 20-HETE formation and blockade of FFAR1 in islets inhibits GSIS. In islets from type-2 diabetic humans and mice, glucose-stimulated 20-HETE formation and 20-HETE-dependent stimulation of GSIS are strongly reduced. We show that 20-HETE is an FFAR1 agonist, which functions as an autocrine positive feed-forward regulator of GSIS, and that a reduced glucose-induced 20-HETE formation contributes to inefficient GSIS in type-2 diabetes.

## Abstract (web summary)

FFAR1 receptor is highly expressed in beta cells and its activation has been suggested as therapy against type-2 diabetes. Here, Tunaru et al. show that 20-hydroxyeicosatetraenoic acid, produced within the islets upon glucose stimulation, acts in an autocrine manner to stimulate insulin secretion via FFAR1 activation.

## Introduction

Insulin is the only hormone able to lower blood glucose levels. In order to maintain glucose homeostasis, insulin secretion from β-cells of pancreatic islets is subject to a well-coordinated regulation. Secretion of insulin is primarily controlled by the entry of glucose into β-cells. However, glucose-stimulated insulin secretion (GSIS) is modulated by multiple factors including mediators of the autonomous nervous system as well as various hormones and nutrients^1^. Most of these molecules act through G-protein-coupled receptors expressed by β-cells^2^. Among them is FFAR1, a G_q_/G_11_-coupled receptor which shows high expression in β-cells and can be activated by various long- and medium-chain free fatty acids (FFAs)^3–6^. Shortly after the discovery of FFAR1, synthetic agonists of the receptor have been developed which promote GSIS, and FFAR1 is now an established target for drugs used to increase insulin secretion in type-2 diabetes^7, 8^.

Despite the role of FFAR1 as a therapeutic drug target and the rapid development of synthetic FFAR1 agonists, the physiological function of FFAR1 in regulating insulin secretion from β-cells remains unclear^9, 10^. In transgenic mice with β-cell specific overexpression of FFAR1, acute stimulation of insulin secretion by a bolus injection of glucose was increased^11^ whereas it was impaired in Ffar1-deficient mice kept on a high-fat diet^12^. Insulin secretion in response to a sustained glucose stimulation in hyperglycemic clamp experiments was even more strongly reduced in Ffar1-deficient mice both under fasted and fed conditions^13^. These data indicate that FFAR1 plays an important role in the regulation of insulin secretion by glucose under physiological and pathological conditions, but it is unclear which physiological ligands are responsible for FFAR1-dependent regulation of insulin secretion.

Based on earlier data, which showed that the artificial lowering of FFA plasma levels resulted in reduced GSIS, whereas elevation of plasma FFA levels increased GSIS^14–18^, it was assumed that FFAR1 functions as a receptor for dietary FFAs^19^. This notion was supported by the observation that in vivo infusion of dietary FFAs as well as in vitro exposure of pancreatic islets to palmitate potentiated GSIS, and these effects were reduced by about 50% in the absence of FFAR1, while the remaining half of this GSIS-promoting effect of FFAs appears to require FFA metabolism to long-chain co-enzyme A esters^12, 20–25^. Since dietary FFAs were always added exogenously, it remains unclear whether dietary FFAs regulate insulin secretion through FFAR1 under physiological and pathophysiological conditions. In addition, dietary FFAs are strongly bound to plasma proteins, and their free circulating levels appear to be rather low to activate FFAR1^9, 10^. Finally, physiological variations of plasma FFA levels, which are primarily the result of adipocyte lipolytic activity, are rather inversely correlated with insulin secretion as lipolysis and FFA plasma levels decrease postprandially when insulin levels go up while their levels increase during starvation when insulin levels are low.

We therefore searched for other endogenous fatty acid ligands of FFAR1 which promote insulin secretion through FFAR1 under physiological conditions. Here we show that glucose promotes the formation of 20-hydroxyeicosatetraenoic acid (20-HETE) in pancreatic islets and that 20-HETE activates FFAR1 with higher efficacy than dietary FFAs. In addition, we provide evidence that 20-HETE acting through FFAR1 mediates a positive feedback regulation during GSIS, a mechanism which is impaired under type-2-diabetic conditions in mice and humans.

## Results

### 20-HETE is a full agonist of FFAR1

**Fig. 1:**
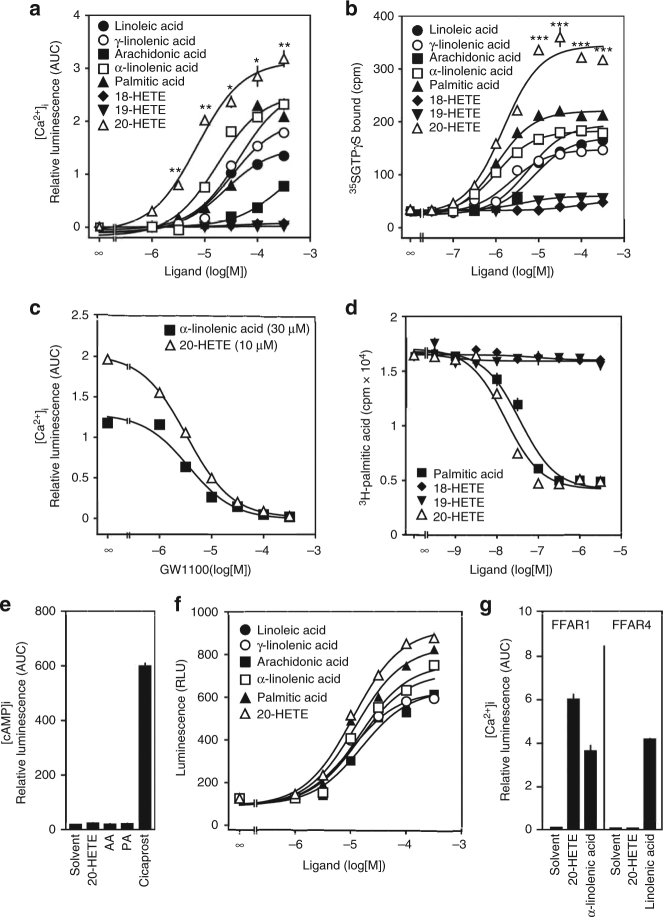
20-HETE is a full agonist of FFAR1. **a** Effect of increasing concentrations of the indicated lipids on [Ca^2+^]_i_ in COS-1 cells transfected with cDNA encoding FFAR1 together with a Ca^2+^-sensitive bioluminescent fusion protein (G5A). **b** Effect of increasing concentrations of the indicated lipids on ^35^S-GTPγS binding to membranes prepared from COS-1 cells expressing FFAR1. **c** Effect of increasing concentrations of the FFAR1 antagonist GW1100 on 20-HETE- and α-linolenic acid-induced increase in [Ca^2+^]_i_ in COS-1 cells. **d** ^3^H-palmitic acid competition binding study with FFAR1 expressed by COS-1 cells. Competition with unlabeled palmitic acid (filled squares) and the indicated lipids is shown. **e** Effect of 30 μM 20-HETE and related lipids (arachidonic acid (AA) and palmitic acid (PA)) on intracellular cAMP levels in COS-1 cells expressing FFAR1 and a cAMP-sensitive bioluminescence probe. As control, COS-1 cells transfected with prostacyclin receptor (IP) were exposed to 1 μM cicaprost (right column). **f** Effect of increasing concentrations of the indicated lipids on β-arrestin recruitment to FFAR1 using the TANGO assay system as described in Material and Methods. **g** Effect of 20-HETE (30 µM) on FFAR1 and FFAR4 heterologously expressed in COS-1 cells together with a cytosolic Ca^2+^-sensitive bioluminescence probe (G5A) as described in Material and Methods. Functionality of the receptors was assessed by exposure to the known agonists α-linolenic or linoleic acid (30 μM). Shown are mean values ±s.e.m., *n* = 9, *P ≤ 0.05; **P ≤ 0.01 (compared to α-linolenic acid (**a**)); ***P ≤ 0.001 (compared to palmitic acid (**b**)) (Student’s two-tailed *t*-test)

In a screen for lipid mediators which are able to activate FFAR1, we found that 20-HETE was significantly more efficacious than dietary FFAs including linoleic and pinolenic acid as well as palmitic and α-linolenic acid in activating FFAR1 when tested for their ability to induce FFAR1-mediated induction of Ca^2+^ transients in intact cells (Fig. 1a). Very similar data were obtained when FFAR1-mediated increases in GTPγS binding were determined in plasma membranes (Fig. 1b). 20-HETE was also slightly more potent than dietary FFAs with regard to the induction of FFAR1-mediated effects (Fig. 1a, b). In contrast to 20-HETE, arachidonic acid was less active, and 18-HETE as well as 19-HETE showed no activity on FFAR1 (Fig. 1a, b). The FFAR1 antagonist GW1100^26^ concentration-dependently inhibited α-linolenic acid as well as 20-HETE-induced elevation in [Ca^2+^]_i_ in cells transfected with FFAR1 (Fig. 1c), confirming that the effect of 20-HETE is mediated by the receptor. In addition, 20-HETE was able to displace ^3^H-palmitic acid from the FFAR1 receptor expressed in COS-1 cells whereas 18- and 19-HETE, which were inactive in cellular FFAR1 activation assays, were unable to compete with ^3^H-palmitic acid (Fig. 1d). Recent data indicate that FFAR1 can not only signal through G_q_/G_11_ but also through G_s_ and β-arrestin, depending on the ligand^27, 28^. Similar to dietary FFAs, 20-HETE acting through FFAR1 did not lead to activation of G_s_ (Fig. 1e) but was able to recruit β-arrestin, although with relatively low potency (Fig. 1f). Interestingly, 20-HETE at 30 μM had no activity on FFAR4 (GPR120), a FFA receptor activated by dietary long-chain fatty acids^29^ (Fig. 1g). We conclude that 20-HETE is a specific orthosteric agonist of FFAR1, which shows significantly increased efficacy compared to other long-chain fatty acids described as FFAR1 agonists so far.

### 20-HETE stimulates GSIS via FFAR1

**Fig. 2:**
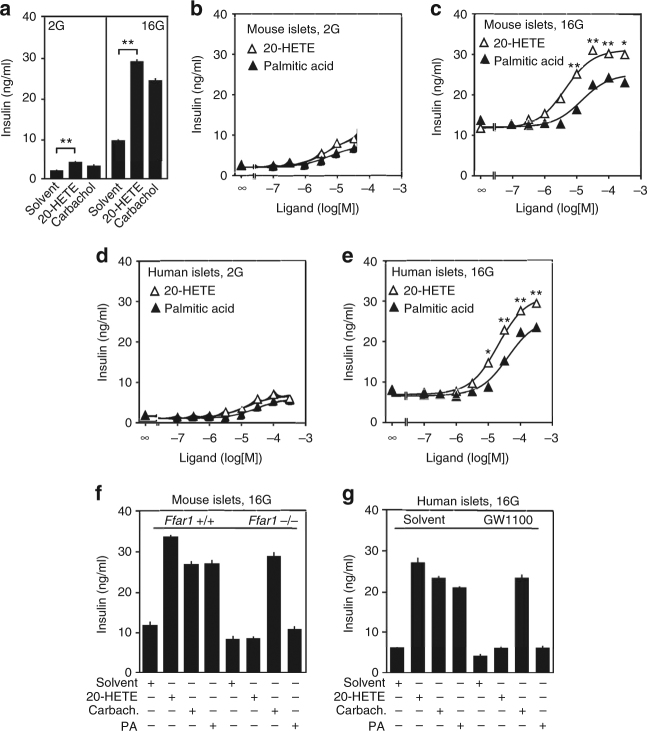
Stimulation of GSIS by 20-HETE in mouse and human islets is mediated by FFAR1. **a** Effect of 20-HETE (10 µM) and carbachol (10 µM) on insulin secretion from murine islets in the presence of low (2.8 mM; 2G) and high glucose concentrations (16.7 mM; 16G). **b**–**e** Effect of 20-HETE and palmitic acid at increasing concentrations on the release of insulin from mouse (**b**, **c**) and human (**d**, **e**) islets in the presence of 2.8 mM (2G) (**b**, **d**) or 16.7 mM (16G) glucose (**c**, **e**). **f**, **g** Effect of 30 μM of 20-HETE, carbachol (carbach.), and palmitic acid (PA) on the release of insulin from murine (**f**) and human (**g**) islets. Murine islets were prepared from wild-type (*Ffar1*^+/+^) or Ffar1-deficient mice (*Ffar1*^−/−^), and human islets were co-incubated with 30 μM of FFAR1 antagonist GW1100 or solvent. Shown are mean values ±s.e.m., *n* = 9; *P ≤ 0.05; **P ≤ 0.01 (Student’s two-tailed *t*-test)

We then tested in isolated mouse and human islets, whether 20-HETE was able to increase insulin secretion at low and high glucose concentrations. 20-HETE promoted GSIS in islets to a similar degree as the muscarinic receptor agonist carbachol (Fig. 2a). 20-HETE concentration-dependently promoted GSIS with an EC50 of about 10 µM, being slightly more potent than palmitic acid (Fig. 2b–e). The effect of 20-HETE was mediated by FFAR1, since in islets from Ffar1-deficient mice 20-HETE had no effect on GSIS while the effect of carbachol was unchanged (Fig. 2f). In addition, 20-HETE-induced stimulation of GSIS in human islets was blocked by GW1100 (Fig. 2g). The maximal effect of 20-HETE on GSIS was significantly higher than the maximal effect of palmitic acid (Fig. 2c, e), which corresponds to the higher efficacy of 20-HETE-induced FFAR1 activation (Fig. 1a, b).

### Glucose promotes 20-HETE synthesis in pancreatic islets

**Fig. 3:**
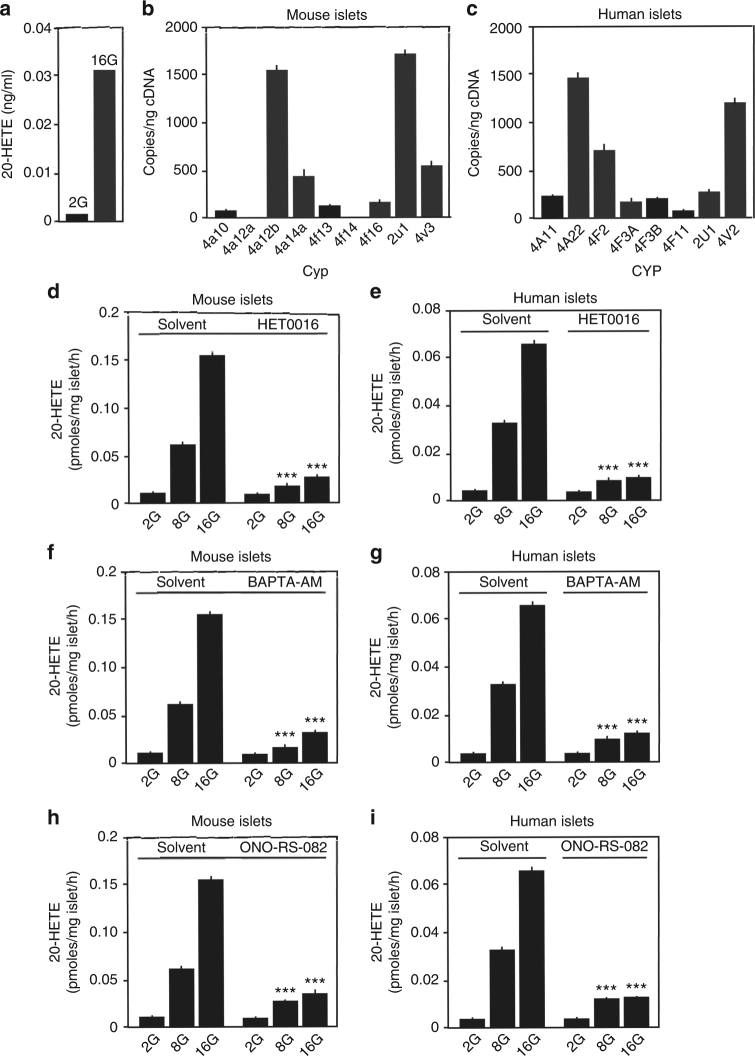
Glucose induces 20-HETE synthesis and release. **a** Mass-spectrometric determination of 20-HETE concentration in supernatants from isolated mouse islets incubated for 2 h in HBSS containing 2.8 mM (2G) and 16.7 mM (16G) glucose (*n* = 1). **b**, **c** Expression of CYP450 ω-hydroxylases involved in 20-HETE synthesis in mouse (**b**) and human pancreatic islets (**c**). **d**–**i** Effect of 30 µM HET0016 (**d**, **e**), 10 µM BAPTA-AM (**f**, **g**), and 30 µM ONO-RS-082 (**h**, **i**) on the formation of 20-HETE in mouse (**d**, **f**, **h**) and human (**e**, **g**, **i**) islets kept in 2.8 (2G), 8.35 (8G), and 16.7 mM (16G) of glucose. Shown are mean values ± s.e.m., *n* = 3 (**b**, **c**), *n* = 9 (**d**–**i**); ***P ≤ 0.001 (compared to solvent) (Student’s two-tailed *t*-test)

Plasma levels of 20-HETE in humans and mice are in the lower nanomolar range^30, 31^, a concentration far too low to activate FFAR1. We therefore tested whether pancreatic islet cells produce 20-HETE which could act locally. We found that isolated murine pancreatic islets produce considerable amounts of 20-HETE, and observed that the amount of 20-HETE increased more than 10-fold in response to a high glucose concentration (Fig. 3a). 20-HETE is an eicosanoid metabolite of arachidonic acid, which is produced by cytochrome P450-dependent ω-hydroxylases of the CYP4 and CYP2 families, including CYP4A11, CYP4A22, CYP4F2, CYP4F3, CYP4V2, and CYP2U1 in humans and Cyp4a10, Cyp4a12, Cyp4f13, Cyp4f16, Cyp4v3, and Cyp2u1 in mice^32–37^. Several of these isoforms were found to be expressed in murine and human pancreatic islets, respectively (Fig. 3b, c). Analysis of publicly available data from single-cell transcriptome profiling of mouse and human pancreatic islets showed that human and murine β-cells primarily express CYP4V2, CYP4F3, and CYP2U1^38^ or Cyp4f13, Cyp4f16, Cyp4v3, and Cyp2u1^39, 40^, respectively (Supplementary Fig. 1). *N*-hydroxy-*N′*(4-n-butyl-2-methylphenyl)formamidine (HET0016), an inhibitor of CYP-dependent 20-HETE formation^41–43^, blocked glucose-stimulated 20-HETE release from mouse and human islets (Fig. 3d, e), which is consistent with a role of CYP enzymes in 20-HETE formation. Glucose-stimulated 20-HETE release from pancreatic islets was also strongly inhibited by pre-incubation of islets with BAPTA-AM as well as the PLA_2_-inhibitor ONO-RS-082 (Fig. 3f–i), indicating that it required an increase in [Ca^2+^]_i_ and PLA_2_ activation.

### 20-HETE and FFAR1 mediate a positive feedback regulation of GSIS

**Fig. 4:**
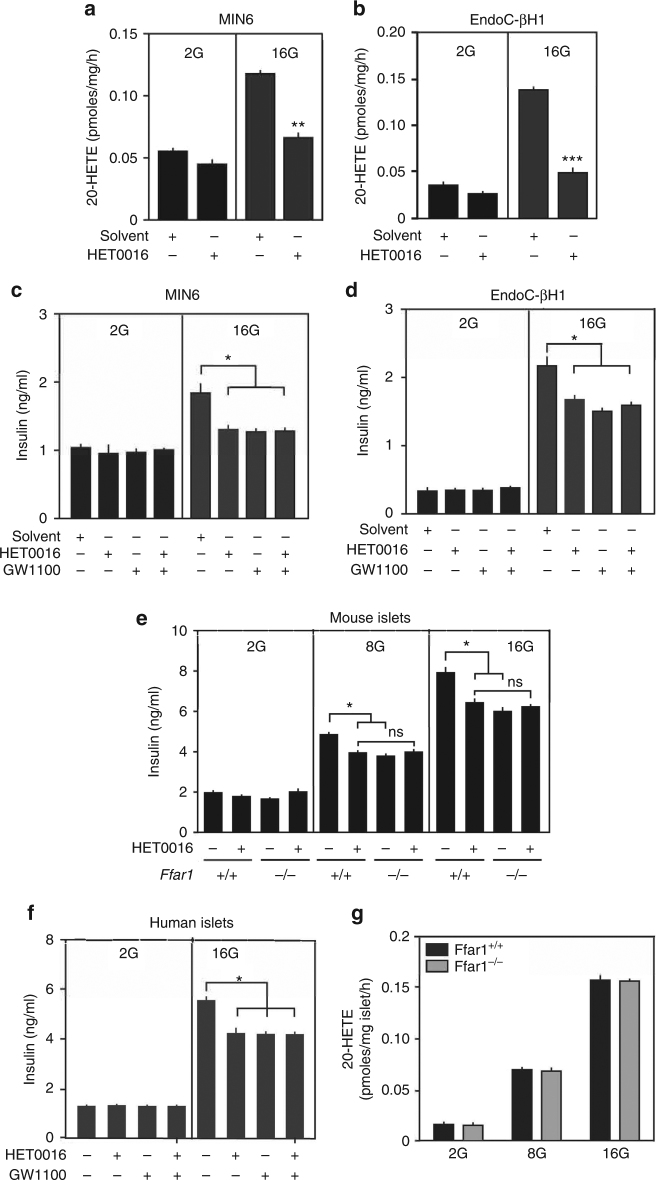
FFAR1 and 20-HETE contribute to GSIS. **a**, **b** Effect of HET0016 on glucose induced release of 20-HETE from MIN6 (**a**) and EndoC-βH1 cells (**b**). **c**, **d** Effect of HET0016 (30 µM) and GW1100 (30 µM) on insulin secretion from MIN6 (**c**) and EndoC-βH1 cells (**d**) kept in 2.8 (2G) and 16.7 mM (16G) of glucose. **e** Effect of HET0016 (30 µM) on insulin secretion from wild-type (*Ffar1*^+/+^) and Ffar1-deficient (*Ffar1*^−/−^) islets kept in 2.8 (2G), 8.35 (8G), and 16.7 mM (16G) of glucose. **f** Effect of HET0016 (30 µM) and GW1100 (30 µM) on insulin secretion from human islets kept in 2.8 (2G) and 16.7 mM (16G) of glucose. **g** Glucose-stimulated 20-HETE release from wild-type (*Ffar1*^+/+^) and Ffar1-deficient (*Ffar1*^−/−^) islets. Shown are mean values ±s.e.m., *n* = 6 (**a**–**d**), *n* = 9 (**e**, **f**), *n* = 3 (**g**); *P ≤ 0.05; **P ≤ 0.01; ***P ≤ 0.001 (Student’s two-tailed *t*-test)

Given that glucose promotes the formation and release of 20-HETE from pancreatic islets and since 20-HETE can stimulate GSIS by its agonistic activity on FFAR1, we reasoned that 20-HETE may be part of a positive feedback loop enhancing GSIS. To test this, we first used the murine and human β-cell lines MIN6^44^ and EndoC-βH1^45^, which express various 20-HETE producing CYP enzymes (Supplementary Fig. 2). Glucose induced in both cells a release of 20-HETE which was blocked by HET0016 (Fig. 4a, b). In both cell lines, GSIS was significantly reduced by HET0016 and by GW1100 in a non-additive fashion (Fig. 4c, d). We then analyzed GSIS from mouse and human pancreatic islets, which were kept in a small volume to allow putative factors, which are released after glucose exposure, to accumulate and to reach sufficiently high concentrations. Again, GSIS in mouse and human islets was significantly reduced in the absence of mouse Ffar1 and after blockade of the human receptor as well as after inhibition of 20-HETE formation by HET0016 in a non-additive fashion (Fig. 4e, f). The amount of 20-HETE released from islets in response to glucose was not affected by loss of Ffar1 (Fig. 4g). These data indicate that CYP-mediated 20-HETE formation and FFAR1 function in series and that 20-HETE mediates a β-cell autonomous positive regulation of GSIS.

### The role of 20-HETE and FFAR1 in diabetic islets

**Fig. 5:**
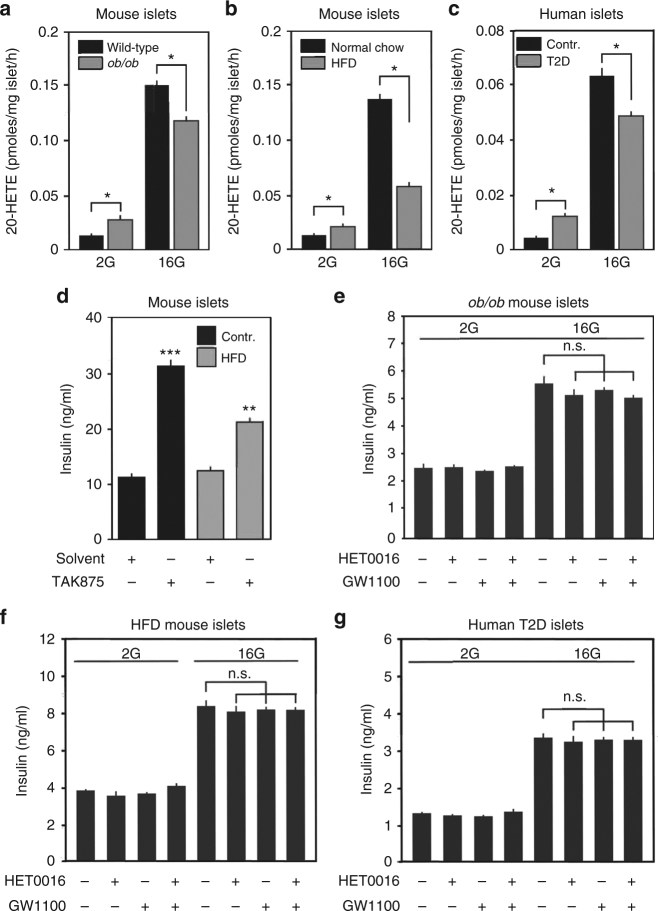
20-HETE/FFAR1-mediated stimulation of GSIS is strongly reduced in type-2 diabetes. **a**–**c** 20-HETE release from islets isolated from lean wild-type and obese leptin-deficient mice (*ob/ob*) at an age of 20 weeks (**a**) from mice fed normal chow or HFD for 23 weeks (**b**) or from non-diabetic (contr.) and type-2 diabetic human donors (T2D) (**c**) in the presence of 2.8 (2G) and 16.7 mM (16G) of glucose. **d** Effect of the specific FFAR1 agonist TAK-875 (30 µM) on GSIS at 16.7 mM of glucose in islets from non-diabetic mice fed normal chow diet (control diet) and from diabetic mice fed HFD for 23 weeks (high-fat diet). **e**–**g** Effect of HET0016 (30 µM) and GW1100 (30 µM) on insulin secretion from murine islets prepared from leptin-deficient mice (*ob/ob*) (**e**), mice fed a HFD (**f**), or from human islets prepared from two type-2 diabetic (T2D) donors (**g**) kept in 2.8 (2G) and 16.7 mM (16G) of glucose. Shown are mean values ± s.e.m., *n* = 6 (**a**–**c**), *n* = 9 (**e**–**g**); *P ≤ 0.05; **P ≤ 0.01; ***P ≤ 0.001 (Student’s two-tailed *t*-test)

In pancreatic islets from aged leptin-deficient mice and from HFD-fed animals, which are obese and type-2-diabetic, as well as in islets from type-2 diabetic patients basal 20-HETE formation was increased (Fig. 5a–c). In contrast, glucose-stimulated 20-HETE formation was decreased in type-2 diabetic mouse and human islets (Fig. 5a–c). In addition to a reduced glucose-stimulated 20-HETE formation in diabetic islets, we found that stimulation of GSIS by the selective FFAR1 agonist TAK-875 was reduced in islets from HFD-fed diabetic mice compared to non-diabetic animals fed a normal chow diet (Fig. 5d). Consistent with an attenuation of 20-HETE/FFAR1-mediated stimulation of GSIS in diabetic islets, we found that inhibition of 20-HETE formation by HET0016 and blockade of FFAR1 by GW1100 reduced GSIS in islets from leptin-deficient and HFD-fed mice to a lesser degree than in normal islets (Fig. 5e, f). In islets from type-2 diabetic patients, both HET0016 and GW1100 were unable to inhibit GSIS (Fig. 5g). This indicates that the 20-HETE-mediated positive regulatory loop is impaired in diabetic islets.

## Discussion

**Fig. 6:**
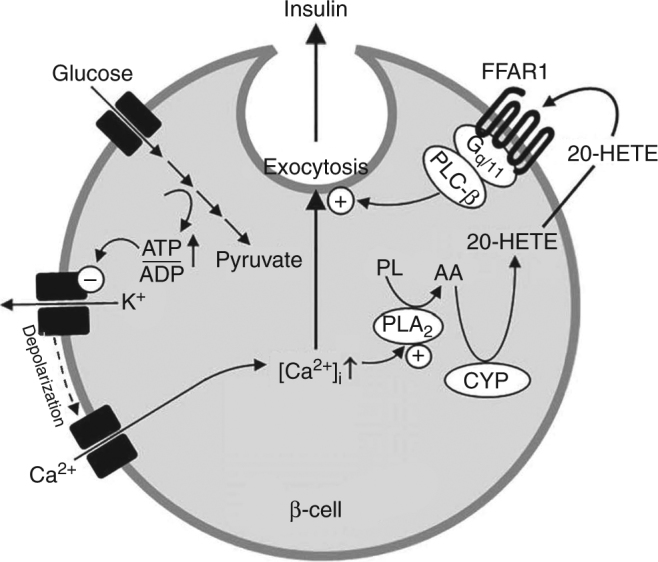
Model of 20-HETE-mediated stimulation of GSIS through FFAR1. Glucose-induced increases in intracellular [Ca^2+^] result in phospholipase A_2_ (PLA_2_) activation. Arachidonic acid (AA) produced by PLA_2_ from phospholipids (PL) is then further converted by CYP ω-hydroxylases to 20-HETE, which in an auto- and/or paracrine fashion activates the G_q_/G_11_-coupled FFAR1 receptor. Downstream signaling induced by activated FFAR1 promotes GSIS

In a search for lipid mediators which function as physiological FFAR1 agonists in the regulation of insulin secretion, we identified 20-HETE as a new regulator of pancreatic β-cells. 20-HETE turned out to be a highly efficacious agonist of FFAR1, which is produced by PLA_2_ and ω-hydroxylases after uptake of glucose by β-cells. Inhibition of 20-HETE formation as well as blockade of the FFAR1 receptor reduced GSIS. This indicates that 20-HETE and FFAR1 function together in a positive feedback loop to enhance GSIS (see Fig. 6).

FFAR1 can be activated by multiple medium and long-chain fatty acids. Several reports comparing the agonistic properties of various dietary FFAs on FFAR1 describe differences with regard to their potency, while their efficacy appeared to be similar^3–5^. More recently Christiansen et al.^6^ reported different efficacies for various dietary FFAs with linoleic and pinolenic acid being most efficacious. We show here that 20-HETE activates FFAR1 with a significantly higher efficacy than any dietary FFA including linoleic and pinolenic acid when analyzed in Ca^2+^ mobilizations and GTPγS binding assays as well as in tests to analyze stimulatory regulation of GSIS in isolated mouse and human islets. This supports the relevance of 20-HETE as a physiological and potentially also pathophysiological agonist of FFAR1 and indicates that 20-HETE is a full agonist of FFAR1 whereas dietary FFAs are rather partial agonists.

Under in vivo conditions, insulin secretion induced by a single bolus of glucose was hardly affected in mice with constitutive FFAR1 deficiency^12, 20^. In contrast, sustained elevation of glucose plasma levels, which better mimics a physiological glucose challenge and involves secondary amplification mechanisms of insulin secretion, resulted in an about 60% reduction of insulin secretion in fed and fasted Ffar1-deficient mice^13^. These data clearly indicate an important role of FFAR1 in the stimulatory regulation of GSIS. However, since dietary FFAs are permanently present in the circulation and since changes in their plasma levels are rather inversely correlated with insulin secretion, the observed defects and the difference between the response of Ffar1-deficient animals to acute and prolonged glucose challenges are hard to explain if one assumes that dietary FFAs are the primary FFAR1 agonists in vivo. Our data rather indicate that locally produced 20-HETE mediates the amplification of GSIS via FFAR1. In isolated islets, the 20-HETE release in response to high glucose increased from 0.02 to 0.15 pmoles per islet per hour (see Fig. 4g). Provided that an average mouse pancreatic islet has a volume of about 500 pl^46, 47^ and that its extracellular volume is about 20% of that^48^, 100 pl, the release of 0.15 pmoles of 20-HETE per islet and hour would result in the accumulation of an extracellular 20-HETE concentration of about 25 μM within 1 min, which is sufficient to maximally activate FFAR1. Although, this is just an approximate calculation of the extracellular 20-HETE concentration in pancreatic islets, it demonstrates the plausibility of an autocrine mechanism involving 20-HETE and FFAR1 in the regulation of insulin secretion.

20-HETE is an eicosanoid metabolite of arachidonic acid, which is produced by ω-hydroxylase members of the cytochrome P450-dependent CYP4 and CYP2 families, in particular CYP4A11, CYP4A22, CYP4F2, CYP4F3, CYP4V2, and CYP2U1 in humans and Cyp4a10, Cyp4a12, Cyp4f13, Cyp4f16, Cyp4v3, and Cyp2u1 in mice^32–37^, of which we found several to be expressed in pancreatic islets and β-cells. It is well known that exposure of pancreatic β-cells to glucose induces a phospholipase A_2_ (PLA_2_)-mediated formation of arachidonic acid, the precursor of 20-HETE^49, 50^, and glucose-stimulated PLA_2_ activation is required for GSIS^51, 52^. We found that glucose-stimulated 20-HETE release from pancreatic islets was strongly inhibited by pre-incubation of islets with BAPTA-AM as well as the PLA_2_-inhibitor ONO-RS-082. We therefore conclude that glucose induces 20-HETE formation through Ca^2+^- and PLA_2_-dependent formation of arachidonic acid from membrane phospholipids. Arachidonic acid is then further metabolized to 20-HETE by Cyp ω-hydroxylases (see Fig. 6). Systemic elevation of 20-HETE levels in transgenic mice with constitutive CYP4F2 overexpression in renal tubular cells has been reported to result in hypertension and hyperglycemia including attenuated GSIS^53^. It is difficult to interpret these data since unphysiological long-term elevation of systemic 20-HETE levels leading to hypertension is likely to induce multiple secondary effects which can reduce GSIS, but it is also possible that it results in desensitization of the 20-HETE-mediated regulation of GSIS described here which would explain the attenuation of GSIS.

FFAR1 is also expressed in various other cells including enteroendocrine cells where it is believed to mediate FFA-induced release of incretins such as glucagon-like peptide-1 and gastric inhibitory polypeptide^28, 54, 55^. It is tempting to speculate that 20-HETE or other hydroxylated non-dietary fatty acids with agonistic activity on FFAR1 play a role in FFAR1-mediated regulation of intestinal epithelial functions. In fact, the hydroxylated gut microbial linoleic acid metabolite, 10-hydroxy-cis-12-octadecenoic acid, was recently shown to ameliorate intestinal epithelial barrier impairment in part by acting as an agonist of FFAR1^56^. FFAR1 is also expressed by pancreatic α-cells and can mediate FFA-induced glucagon secretion^57–59^. The physiological function of this regulation is unclear but it appears possible that 20-HETE produced by β-cells in the presence of high glucose concentrations may act in a paracrine fashion to modulate glucagon secretion from α-cells. Several reports have recently shown that FFAR1 is expressed in various tumors and that it may influence tumor growth and progression^60^. Since 20-HETE is produced by many tumors and has been shown to promote tumor growth and metastasis^61^, it will be interesting to explore a potential role of 20-HETE/FFAR1-mediated signaling in tumor progression

20-HETE has multiple effects in the mammalian organism but is best known for its role in cardiovascular diseases in particular arterial hypertension^62^. 20-HETE stimulates vascular smooth muscle contraction and endothelial cell dysfunction, which promote vascular diseases including hypertension and atherosclerosis^33^. It is unlikely that FFAR1 is involved in these effects as the receptor is not expressed in endothelial cells or vascular smooth muscle cells^63, 64^. Instead, GPR75, which can be activated by 20-HETE and appears to be expressed in vascular cells, is a likely candidate to mediate cardiovascular effects of 20-HETE^65^.

FFAR1 is an important target for antidiabetic drugs, but its physiological function remains unclear. In this report, we provide evidence that 20-HETE is an efficacious endogenous agonist of FFAR1, which after its glucose-induced formation in β-cells is released and mediates a positive feed-forward  mechanism to promote GSIS. In islets from type-2 diabetic mice and humans the glucose-stimulated 20-HETE release and the 20-HETE-mediated part of GSIS were reduced. Defects in glucose-induced 20-HETE formation and release or in downstream signaling events may therefore be responsible for inefficient GSIS, and enhancement of glucose-stimulated 20-HETE formation, release, and function could be a strategy to improve GSIS in diabetic patients.

## Methods

### Reagents

20-HETE and related HETEs, PGE_2_, PGD_2_, U46619, cicaprost, cloprostenol, α-linolenic, linoleic, γ-linolenic acids, GW1100, HET0016, and TAK-875 were from Cayman Chemicals. Carbachol, propionic, palmitic, and arachidonic acid were from Sigma-Aldrich. Lipofectamine 2000 was from Life Technologies.

### LC-MS/MS analysis

Quantification of 20-HETE was done in principle as described previously^66^. In brief, 200 μl supernatant were spiked with the corresponding deuterated internal standards and extracted by liquid–liquid extraction using ethyl acetate. Analytes were separated using a Gemini NX C18 RP-LC-column (150 mm × 2 mm I.D., 5 µm particle size and 110 Å pore size from Phenomenex, Aschaffenburg, Germany) under gradient conditions with water and acetonitrile as mobile phases, both containing 0.01% ammonia solution. The LC system was coupled to a mass spectrometer 5500 QTrap (Sciex, Darmstadt, Germany) equipped with a Turbo-V-source operating in negative electrospray ionization mode. Data acquisition was done using Analyst Software V 1.6 and quantification was performed with MultiQuant Software V 3.0 (Sciex, Darmstadt, Germany) employing the internal standard method (isotope dilution mass spectrometry).

### Isolated islet studies

Mouse pancreatic islets were isolated by collagenase digestion as previously described^67^. In short, islets were prepared by collagenase P digestion, handpicked, and then incubated in a humidified atmosphere in RPMI 1640 culture medium (Life Technologies) supplemented with 10% (vol/vol) FBS, 100 U/ml penicillin, and 100 U/ml streptomycin. To measure insulin secretion, islets were preincubated for 30 min at 37 °C in a Krebs-Ringer bicarbonate buffer (KRB) composed of 115 mM NaCl, 4.7 mM KCl, 1.2 mM KH_2_PO_4_, 1.2 mM MgSO_4_, 2.56 mM CaCl_2_, 20 mM NaHCO_3_, 10 mM HEPES, and supplemented with 5 mM glucose and 0.1% bovine serum albumin (BSA, fatty acid-free). After washing, 6–8 islets were incubated at 37 °C for 1 h in 20 or 200 µl KRB supplemented with the indicated glucose concentration and other test compounds as indicated. Insulin concentration was determined in the supernatant by enzyme-linked immunosorbent assay (ELISA) (Millipore), following the manufacturer’s protocol.

Freshly isolated human pancreatic islets were purchased from Prodo Laboratories. Islets were from five donors (3 non-diabetic donors (age 27, female, with a body mass index (BMI) of 27.7, non-diabetic; age 37, female, with a BMI of 22.9, non-diabetic; age 23, male, with a BMI of 24.8, non-diabetic) and two diabetic donors (age 56, female, with a BMI of 29 diagnosed for 7 years with type-2 diabetes and age 51, male, with a BMI of 35.6 and a history of type-2 diabetes for 4 years) and were shipped in a cooling package. After arrival islets were immediately cultured in Prodo standard islet medium (PIM(S)) according to the provider’s instructions. Formal consent by the donors and ethical approval was obtained. Insulin secretion assays were performed on the second day after arrival of islets using a human insulin ELISA kit from Sigma-Aldrich and the same protocol as described for murine islets.

### 20-HETE determination

Isolated pancreatic islets from mice and human (6 islets/sample) were preincubated for 30 min at 37 °C in HBSS containing 1.8 mM Ca^2+^ and 5 mM glucose. After that, they were stimulated with 100 µl of HBSS containing Ca^2+^ and different glucose concentrations as indicated for 2 h at 37 °C under gentle shaking (100 rpm). To determine 20-HETE release from MIN6 and EndoC-βH1 cells, 50,000 cells were seeded in each well of a 96-wells plate. Forty-eight hours later the medium was changed to HBSS containing 1.8 mM Ca^2+^ and 5.5 mM glucose and were allowed equilibrating for 1 h at 37 °C. Thereafter, they were stimulated in 100 µl HBSS including 1.8 mM Ca^2+^ and indicated glucose concentrations and test compounds for 2 h at 37 °C. 20-HETE secretion in the medium was determined by using an ELISA assay kit (Detroit R&D, Inc.) following the manufacturer’s instructions.

### Cell transfection and determination of [Ca^2+^]_i_

COS-1 cells, obtained from ATCC, were seeded in 96-well plates with white walls and transparent bottom and transfected with plasmids containing cDNAs encoding a calcium-sensitive bioluminescent fusion protein consisting of aequorin and GFP^68^ and the indicated receptors by using Lipofectamine 2000 (Life Technologies) following the manufacturer’s instructions. Forty-eight hours later, cells were loaded with 5 µM coelenterazine h (Promega) in HBSS containing 1.8 mM calcium and 10 mM glucose for 2 h at 37 °C. Measurements were performed using a luminometric plate reader (Flexstation 3; Molecular Devices). The area under each calcium transient was calculated by using SoftMaxPro software and expressed as area under the curve (AUC).

### Intracellular cAMP determination

COS-1 cells were transiently transfected with FFAR1 and a plasmid encoding a cytosolic cAMP-sensitive bioluminescent probe (GloSensor^TM^ cAMP Assay; Promega). Forty-eight hours after transfection, cells were incubated in HBSS containing 1.8 mM Ca^2+^, 10 mM glucose, and 2% (v/v) of GloSensor^TM^ cAMP reagent for 2 h at room temperature. Intracellular cAMP was determined by using a luminometer plate reader following stimulation with ligands for 15 min. Light generated during agonist stimulation was expressed as AUC.

### Beta-arrestin assay

To determine ligand-induced interaction of FFAR1 and β-arrestin, we used the TANGO assay^69^. The FFA1-Tango plasmid, a gift from Bryan Roth, was obtained from Addgene (plasmid # 66280). HTLA cells^70^ were transfected with FFAR1-Tango in 96-well plates with white walls and transparent bottom. Thereafter, cells were stimulated for 16 h with the indicated ligands in sterile HBSS buffer containing 1.8 mM Ca^2+^ and 10 mM glucose. Next day, the stimulation buffer was removed and replaced by 200 μl assay buffer containing 10% Bright-Glo reagent (Promega). After 20 min incubation at room temperature, luminescence was counted in a plate reader and expressed as relative luminescence units.

### Radioligand binding assay

To determine equilibrium binding of [9,10-^3^H(N)]-palmitic acid (Hartmann-Analytik, 27 Ci/mmol) to FFAR1 receptor, COS-1 cells seeded in 24-well plates were transfected with an eukaryotic expression plasmid carrying the FFAR1 cDNA. Forty-eight hours later cells were rinsed with ice-cold binding buffer (PBS + 0.5% fatty acid-free BSA) and competition binding assay was performed by incubating transfected cells in binding buffer containing 100 nM ^3^H-palmitic acid and the indicated compounds for 90 min at +4 °C. Binding reaction was stopped by three washing steps with ice-cold binding buffer followed by lysis in 0.1% (v/v) Triton X-200 and 2 N NaOH. Cell lysates were transferred to vials containing scintillation fluid (Ultima-Gold, Perkin Elmer) and the radioactivity was measured with a scintillation counter (Hidex 300SL).

### GTPγS binding assay

To determine FFAR1-mediated G-protein activation, membranes from COS-1 cells transfected with a eukaryotic expression vector carrying cDNA encoding FFAR1 or with empty vector were prepared. Membranes (50 μg of membrane protein) were incubated for 60 min at 35 °C in a total volume of 250 μl of buffer containing 100,000 cpm (0.2 nM) of [^35^S]GTPγS, 1 mM EDTA, 5 mM MgCl_2_, 1 mM dithiothreitol (DTT), 100 mM NaCl, 10 μM GDP, and 50 mM Tris-HCl (pH 7.4). Incubation was terminated by filtration over GF-B glass fiber filters (Wathman) and three washing steps with ice-cold buffer (50 mM Tris-HCl, pH 7.4; 5 mM MgCl_2_ and 0.02% CHAPS). Bound GTPγS was measured by a scintillation counter (Hidex 300SL).

### Cell lines

MIN6 cells^44^ (from the American Tissue Culture Collection (ATCC)) and EndoC-βH1^45^ cells (kindly provided by P. Ravassard and R. Scharfmann (Inserm, Paris, France)) were cultured in Dulbecco's modified Eagle's medium supplemented with 25 mM glucose (MIN6 cells) or with 5.5 mM glucose (EndoC-βH1 cells) supplemented with 2 mM l-glutamine, 1 mM sodium pyruvate, 15% FBS, 50 µM 2-Mercaptoethanol and 100 U/ml penicillin and 100 mg/ml streptomycin (Gibco), 10 mM nicotinamide (Sigma-Aldrich), 5.5 µg/ml transferrin (Sigma-Aldrich), 6.7 ng/ml sodium selenite (Sigma-aldrich) (EndoC-βH1 cells). Cells were tested negative for mycoplasma contamination before experiments.

To determine glucose-induced insulin secretion from MIN6 or EndoC-βH1 cells, 50,000 cells/well were seeded in 96-well plates. Forty-eight hours later, medium was changed to KRB buffer supplemented with 5.5 mM glucose and equilibrated for 1 h at 37 °C. Following the equilibration, cells were stimulated in 20 µl fresh KRB medium containing the indicated glucose concentrations and other test compounds for 1 h at 37 °C. Insulin concentration was determined in the supernatant by ELISA (Milipore) following the manufacturer’s protocol.

### RNA isolation and quantitative RT-PCR

RNA isolation and reverse transcription from mouse and human pancreatic islets were performed by using RNAeasy kit (Qiagen) and ProtoScript II First Strand cDNA Synthesis Kit (New England Biolabs) according to the manufacturer’s instructions. Quantitative RT-PCR was done with primers designed with the Roche’s online tool and a Universal Probe Library assay (Roche). Sequences of primers are given in Supplementary Table 1.

### Genetic mouse models

FFAR1-deficient mice have been described elsewhere^24^ and were kindly provided by Helena Edlund and Pär Steneberg (Umeå Centre for Molecular Medicine, Umeå, Sweden). Leptin-deficient mice (*ob/ob*) were obtained from the Jackson Laboratory (stock number: 000632). Maintenance of the animals and animal experiments were in agreement with the German animal welfare legislation (approval obtained by the Max Planck Institute for Heart and Lung Research from the Regierungspräsidium Darmstadt (Germany)). The mice used in this study were group-housed in ventilated cages (Techniplast Greenline) containing nesting material. They were kept under 12-h light/12-h dark cycle at controlled ambient temperature of 23 °C, 55% relative humidity with free access to water, and fed ad libitum. For studies in type-2 diabetic mice, 6-week-old male mice were fed with high-fat diet containing 54% (metabolizable energy) fat which is equivalent to 30% (wt/wt) fat (ssniff GmbH) for 17 weeks. In some cases C57BL/6J mice on D12492 (60 kcal% fat) and control mice maintained on D12450B (10 kcal% fat) for 23 weeks were purchased from JAX.

### Statistics

Trial experiments or experiments done previously were used to determine sample size with adequate statistical power. The investigator was blinded to the group allocation and during the experiment. Data presented are expressed as mean ± s.e.m. Statistical analyses were performed by using GraphPad Prism software. Unpaired Student’s *t*-test was used to compare two groups.

### Data availability

All relevant data are available from the authors upon reasonable request.

## Electronic supplementary material

Supplementary Information
