# Supplementary material for: 20-HETE promotes glucose-stimulated insulin secretion in an autocrine manner through FFAR1
Source: Nat Commun. 2018 Jan 12;9:177. doi: 10.1038/s41467-017-02539-4 (PMC5766607; doi:10.1038/s41467-017-02539-4)
Supplement: Supplementary file 1 — Supplementary Information [file 41467_2017_2539_MOESM1_ESM.pdf]

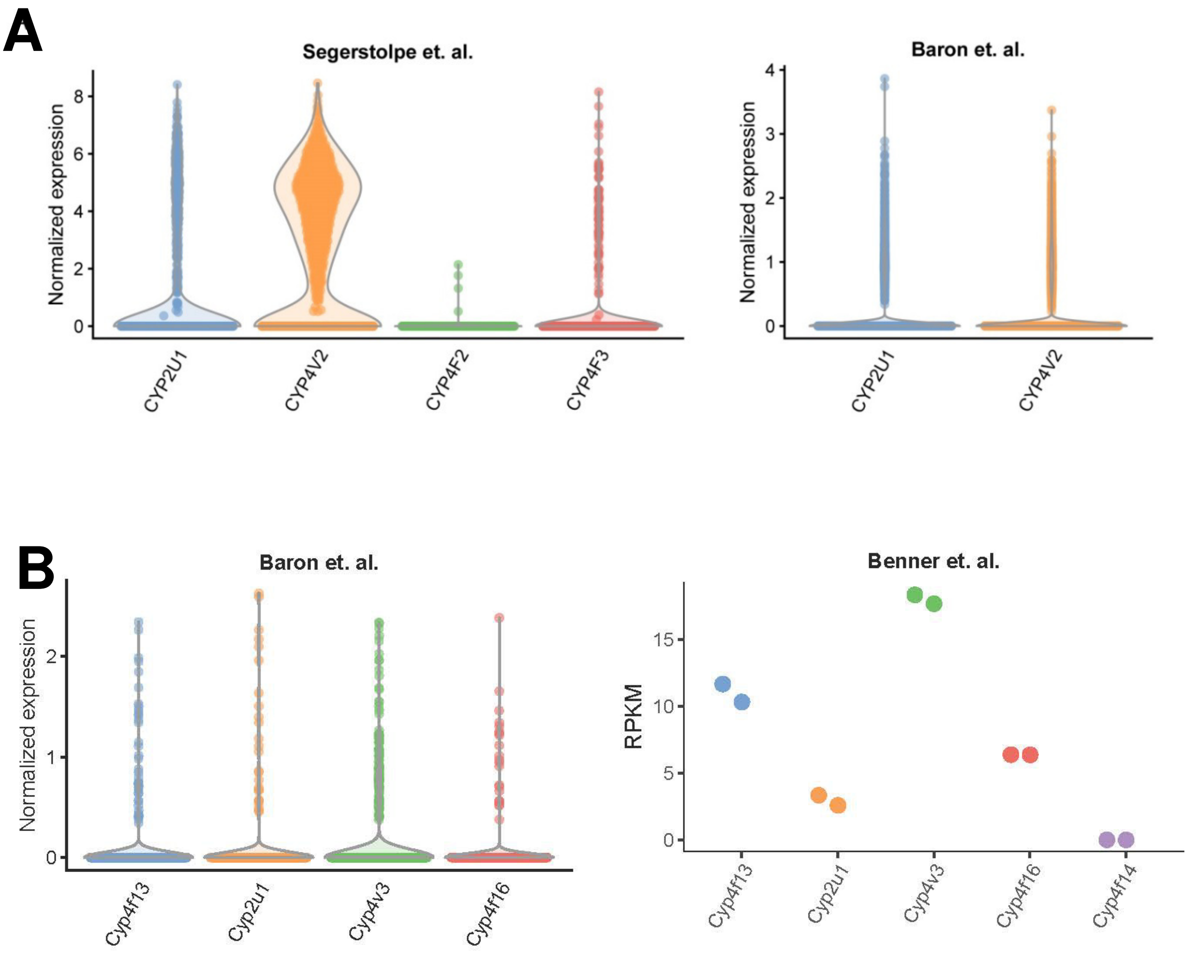

Supplementary Figure 1. Expression of 20-HETE producing CYP450 ω-hydroxylases in human and mouse β-cells. (**A**) Human β-cell single cell expression of selected genes encoding 20-HETE producing CYP450 ω-hydroxylases from Segerstolpe et al. (2016) and Baron et al. (2016). Genes are only shown if expression was detected in at least one cell. (**B**) Expression of selected genes from single mouse β-cells (Baron et al., 2016) or bulk sorted β-cells (Benner et al., 2014). Genes are only shown if expression was detected in at least one cell for data from Baron et al. (2016). Data from Benner et al. (2014) were taken from additional file 12 and were not processed further.

Methods: Raw count data were downloaded from GEO and ArrayExpress (Accession numbers GSE84133 and E-MTAB-5061, respectively). We created three data objects in R using the SingleCellExperiment function from the *scater* package (version 1.5.16) for mouse and human donors from Baron et al. (2016) and for human donors from Segerstolpe et al. (2016). All data objects were subjected to a gene-filtering step to only retain genes that were detected in at least 4 cells. Expression was then normalized using the computeSumFactors method from the *scran* package using deconvolution within donors.

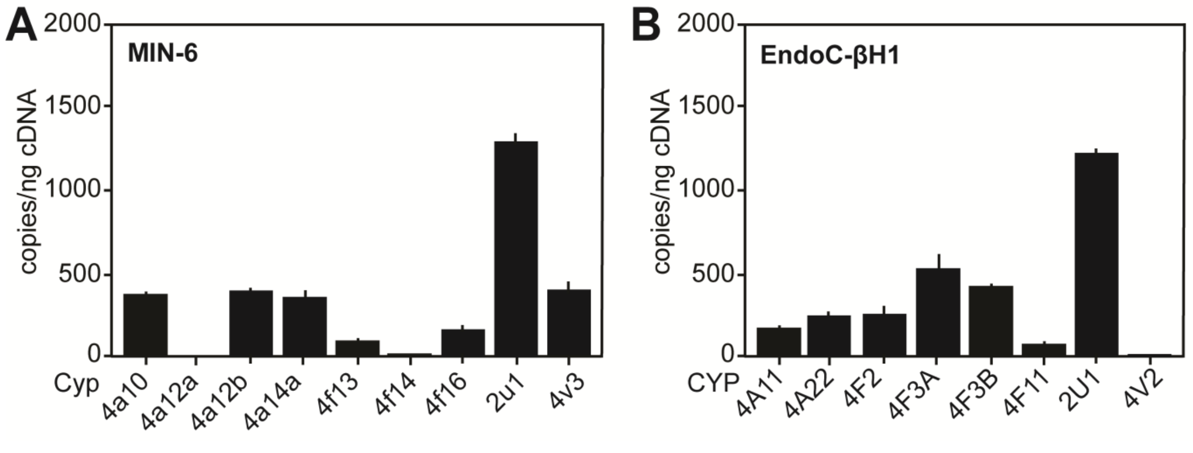

Supplementary Figure 2. Expression of various 20-HETE producing CYP450 ω-hydroxylases in MIN-6 and EndoC-βH1 cells. (**A**, **B**) Expression of CYP450 ω-hydroxylases involved in 20-HETE synthesis MIN-6 (A) and in EndoC-βH1 cells (B). Shown are mean values ± s.e.m..

Supplementary Table

Table 1. Primers used for quantitative RT-PCR

Gene target	Sense primer	Antisense primer
**mouse**		
Cyp4a10	gttccagcacaggaggatg	cagccgttcccatttgtc
Cyp4a12a	atccttctcgatttgcacca	caaactgtttcccaatgcag
Cyp4a12b	acggaaatcatggcagactc	tcatcaaggtgatgtgttgga
Cyp4a14a	gccattctcaggaggatcaa	tctggcagcaattcaaagc
Cyp4f13	ctcctgatgagtgctggtga	ttcaggatgtcaaagtgaaagg
Cyp4f14	tctggttattcccctcacca	ttgggtgaagaggtacaggagt
Cyp4f16	cgatagggaggcaccataga	gtgggaggttacatgccagt
Cyp2u1	gcttcgtcatttcggtctg	acgtatgcaaactcctcgat
Cyp4v3	tttccatggcctagcaattag	ctttggatgctgattcatgg
Ffar1	aggcgctctcctcacactc	ctagccacattggaggcatt
**human**		
CYP4A11	gtgctgcaccatgagtgtct	ccagagacatcacccagga
CYP4A22	tgcaccatgagtgtctctgtc	agggaggtcacttggaggat
CYP4F2	cattgcaccaaaggacaagtt	caccagcactcagcaggag
CYP4F3A	tgcagaaatgtgtcttcagctt	ggcggcaatatattcactgg
CYP4F3B	cgtctctgcctctctccact	ggtggtgagtgaggtcctg
CYP4F11	ccctgttgacttctctaatctcttc	aaggccagagtaaccgagtg
CYP2U1	cctcatgctggagttgtgtg	gcacaacatcctcacctcaa
CYP4V2	ggcctccagtgcaatcac	aaagcgaaagaccggaaaac
FFAR1	gtgtcacctgggtctggtct	ccagggaggtgttgctgt
